# Preliminary Development of a Database for Detecting Active Mounting Behaviors Using Signals Acquired from IoT Collars in Free-Grazing Cattle

**DOI:** 10.3390/s25103233

**Published:** 2025-05-21

**Authors:** Miguel Guarda-Vera, Carlos Muñoz-Poblete

**Affiliations:** 1 Magíster en Ciencias de la Ingeniería, Universidad de La Frontera, Temuco 4811230, Chile; m.guarda04@ufromail.cl; 2 Departamento de Ingeniería Eléctrica, Universidad de La Frontera, Temuco 4811230, Chile

**Keywords:** estrus in cows, active mounts, IoT collar, IMU, public database, machine learning, cattle, free-grazing

## Abstract

This study presents the development of a database for detecting active mounts, utilizing IoT collars equipped with Inertial Measurement Units (IMUs) installed on eight Holstein Friesian cows, along with video recordings from a long-range PTZ camera mounted in a central pole for event labeling in the natural environment when free grazing. The resulting database comprises 415 labeled events associated with various behaviors, containing acceleration signals in both the Body and World Frame of reference and gyroscope signals. A Support Vector Machine (SVM) algorithm is implemented to evaluate the effectiveness of the dataset in detecting active mounts and to compare training performance using both frames. The algorithm achieves an average F1 Score of 88.6% for the World Frame of reference, showing a significant improvement compared to the algorithm trained with Body Frame (78.6%) when both are trained with the same 112 features. After applying feature selection using Sequential Backward Selection (SBS), the SVM exhibits a minor performance difference between the F1 Score obtained with the two reference frames (89.7% World Frame vs. 91.5% Body Frame). This study provides a public dataset and a replicable methodology, facilitating future research on movement-based behavior classification in cattle.

## 1. Introduction

In recent years, the Internet of Things (IoT) and machine learning (ML) have proven to be effective tools in the agricultural sector, enabling increased productivity and reducing labor dependency [1]. Technologies such as smart collars and machine learning algorithms have emerged as promising solutions for automating the detection of bovine behaviors, marking a key advancement in livestock modernization. According to [2], which reviewed 413 papers on cattle monitoring, the most common types of technology were found to be inertial sensors (37%) and images or videos (35%). Simple classification methods were used in 48% of the papers, while machine learning was employed in 29%. The two most common objectives in PLF (Precision Livestock Farming) papers were determining animal behavior (30%) and animal health (12%).

The type and quantity of movements performed by cattle indicate their current state. For example, the time cows spend lying down can signal changes in comfort, well-being, and health [3]. Certain behaviors must be detected immediately in dairy farms, such as lameness, which can be identified through characteristics like gait symmetry or gait parameters, such as real-time step counting [4].

Heat detection is one of the most critical aspects in this context. According to [5], cited by [6], estrus signs in cattle can manifest in various ways, including increased physical activity and cow restlessness. Often, interruptions in grazing and feeding are observed, along with a reduction in rumination time and decreased milk production. Instead of grazing, the cow tends to move more, exhibiting behaviors such as attempting to mount other cows or allowing itself to be mounted, regardless of its position in the social hierarchy.

In [7], the authors highlight the occurrence of mounts during the estrus period, whether through attempts or acceptance of this action among cattle.

In [8], the scoring scale proposed by [7] is used to quantify estrus through visual observation of herd behavior. According to this scale, the following scores were assigned to observed behaviors: 100 points for standing to be mounted, 45 points for mounting from the front, 35 points for attempting to mount other cows, 15 points for chin resting, 10 points for mounting without standing, 10 points for vulva sniffing, 5 points for restlessness, and 3 points for the Flehmen response. A cow is considered in estrus upon reaching or exceeding 100 points.

In this study, the action of attempting to mount other cows is defined as active mounting.

Although artificial insemination has been established as a routine practice in livestock production systems, it presents lower conception rates compared to natural mating. According to [9], the primary cause of this phenomenon is inadequate heat detection, which prevents artificial insemination from being performed at the optimal time. This deficiency in detection not only affects the effectiveness of the procedure but also reduces the total milk production throughout the animal’s productive life, decreases the number of calves born per cow, increases open days, and raises the replacement rate due to reproductive issues [6]. The longer a cow remains unpregnant after calving, the lower its milk production, resulting in significant economic losses [10].

Traditionally, the heat detection process is carried out through visual observation, a technique that, although cost-effective, is subjective, time-consuming, and prone to errors [11]. This technique becomes even more challenging in countries like Chile, where dairy farms do not operate under a confined system, but cows feed through grazing. Generally, extensive grazing fields imply a considerable distance between observers and the cattle, making adequate inspection difficult and generating a high dependency on workers and weather conditions.

Various monitoring techniques based on Inertial Measurement Unit (IMU) sensors have been implemented to reduce reliance on visual observation by dairy farm personnel. These sensors include three-axis accelerometers, three-axis gyroscopes, and three-axis magnetometers, allowing the measurement of cows’ inertial movement. In [12], waterproof enclosures with IMUs were installed on collars fixed to cows’ necks. Behaviors classified using machine learning models included feeding, resting, lying-down rumination, standing rumination, social licking, head movement, and headbutting other cows.

Similarly, in [13], IMUs were used to classify different types of behaviors, applying supervised automatic classification algorithms such as Support Vector Machine (SVM), Random Forest, and XGBoost. These achieved high performance indicators, obtaining an overall F1 Score of 0.87, with specific results of 0.93 for lying down, 0.85 for walking, 0.94 for ruminating, 0.89 for feeding, 0.86 for standing, 0.93 for drinking, and 0.59 for other activities. This demonstrates that supervised classification algorithms effectively identify cow behaviors, yielding good results in this context.

A representative and robust database and the application of appropriate preprocessing and model evaluation techniques are necessary to achieve good results in movement classification through training models with supervised learning algorithms. In this sense, in [4], the authors highlight the scarcity of public databases in bovine behavior classification, as most existing ones are commercial or private. To address this limitation, they developed CowScreeningDB, a multisensory database designed to detect lameness in dairy cows. The database includes data from 43 cows collected through smartwatches under natural conditions, organized transparently and replicable, and is publicly available in a GitHub repository, facilitating its use in future research. However, this database does not include specific labels for normal walking or lameness events; instead, it provides a continuous collection of approximately 7 h of daily activity data from healthy and lame cows. It is worth noting that data collection was conducted on cows confined to at least 10 square meters.

The literature contains numerous studies analyzing bovine behavior using databases based on inertial movement signals [12,13,14,15,16,17]. However, these studies use accelerations in the Body Frame (BF) instead of the World Frame (WF). Using the WF instead of the BF for motion data analysis offers several advantages. First, it allows acceleration and orientation measurements to be independent of the sensor’s posture or position, facilitating a more consistent interpretation of the data. Additionally, the WF provides a fixed reference relative to the ground, simplifying the analysis of motion patterns in absolute terms and improving individual comparisons. This characteristic is also advantageous for machine learning algorithms, as it reduces variability unrelated to target behaviors. The hypothesis is proposed that using acceleration data in the terrestrial reference frame would improve the performance of a model trained in this frame compared to one trained in the body reference frame, which must be verified through corresponding experimentation.

This work is part of a research project developed at the Universidad de La Frontera, titled “Towards reliable movement identification for dairy cattle based on self-calibration attitude estimate algorithms”, (Fondecyt 1220178 project) which employs IoT collars equipped with IMUs to capture bovine movement signals. These signals are then used to train classification models to identify cows’ movements associated with heat detection and lameness. As part of this project, a previous database [18] was developed, which included accelerations in the BF measured by the MPU9250 IMU, where various events such as walking, resting, trotting, passive mounting, active mounting, and lameness were labeled. In this instance, a new database is developed, adding orientation information in the form of quaternions obtained from the BNO055 IMU, allowing for the transformation of accelerations from the BF to the WF, using the algorithm developed by its manufacturer, Bosch [19].

This work aims to describe the procedure used to generate a public database focused on detecting active cows’ mounts and making it publicly available to keep improving the classification of cows’ mounts in a free-grazing regime. The database includes the full nine-axis signals (3D accelerometers, gyroscopes, and magnetometers) acquired at 10 samples per second from IMUs in IoT collars worn by the cows, with reference frames in both BF and WF. The cow’s movements are manually identified and labeled using video records obtained with a PTZ camera installed in a 9-m post in the center of the grazing fields.

## 2. Materials and Methods

**Figure 1:**
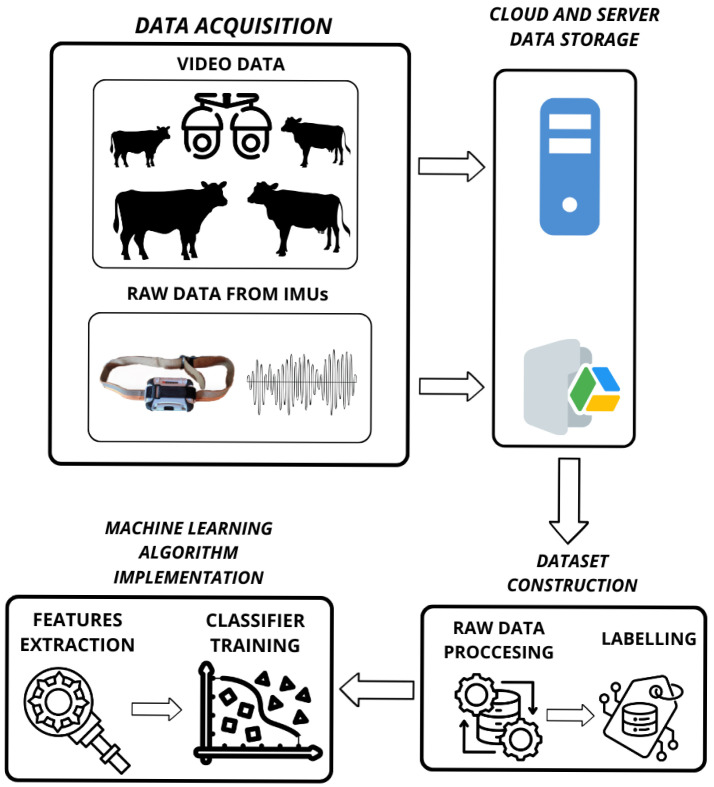
Diagram of the methodology.

This section describes the proposed methodology, illustrated in Figure 1.

### 2.1. Data Acquisition

#### 2.1.1. Field Details

Data are acquired at the Maquehue Experimental Farm, a grazing area managed by the Universidad de La Frontera, located 17 km south of Temuco (Latitude: −38.8379, Longitude: −72.6938). The total surface area is estimated at 250 hectares. Various activities are conducted there, primarily focused on education, research, and commercial production, making it a reference point for the agricultural sector in the Araucanía region.

The terrain’s characteristics and conditions provide an optimal environment for studying the behavior of Holstein Friesian dairy cows. A large herd of cattle is available for the study, grazing in open pastures with access to water through troughs distributed at various points across the field.

**Figure 2:**
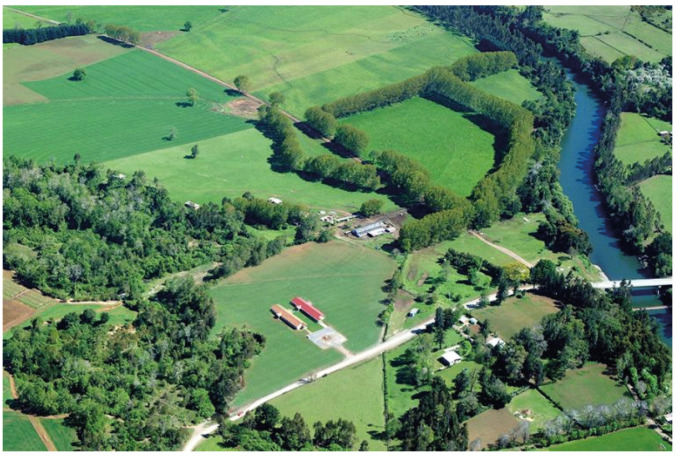
Maquehue Experimental Farm.

The Fondecyt 1220178 project began recording videos and collecting data in May 2022 and has continued. Figure 2 presents an image of the field.

#### 2.1.2. IoT Collar

The wearable IoT device consists of a collar worn by the cows under study. This collar has an ESP32S3 processor on a Wireless Stick Lite V3 (Heltec Automation, Chengdu, China), and IMU sensors: BNO055 (Bosch Sensortec, Kusterdingen, Germany) and MPU9250 (TDK InvenSense, San José, CA, USA), configured to capture acceleration and gyroscope data at a 10 Hz sample rate. The BNO055 sensor is selected for its ability to perform internal orientation calculations using quaternions, allowing for a straightforward transformation of accelerations into the WF, while MPU9250 is a low-cost alternative to obtain accelerations. The IMU data acquired by the collar are locally stored on microSD cards and manually retrieved every 2 weeks when the battery depletes. The IoT collars are monitored regarding location with a GPS and battery status through dashboards in the Grafana platform linked via the LoRaWAN network to the cow’s IoT collars. This protocol has been successfully applied previously in the literature for communication in estrus detection collars for dairy farms, showing a lower energy consumption than other technologies like Sigfox or NB-IoT. Additionally, its link can extend the range to several kilometers, which is crucial for large dairy farms, such as those in free-grazing systems [20].

**Figure 3:**
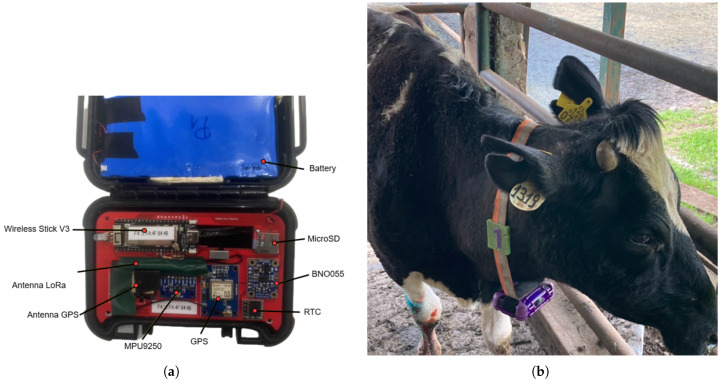
Implementation of the IoT collar: (**a**) Internal structure of the IoT collar. (**b**) Cow wearing the collar.

The prototype is shown in Figure 3a. In Figure 3b, a cow can be seen wearing an IoT collar around its neck.

#### 2.1.3. Video System

Two PTZ video cameras, model IP DAHUA 2MP SD6AL230F-HNI (Dahua Technology, Hangzhou, China), along with a Network Video Recorder (NVR), model DHI-NVR210HS-I (Dahua Technology, Hangzhou, China), complement the system by continuously recording footage to validate the motion data obtained from the collars. These cameras provide a visual perspective essential for manually labeling events. They feature night vision and can monitor the cows up to 500 m. Additionally, using the position data acquired from the collars, the PTZ cameras are automatically controlled to track the animals [21].

**Figure 4:**
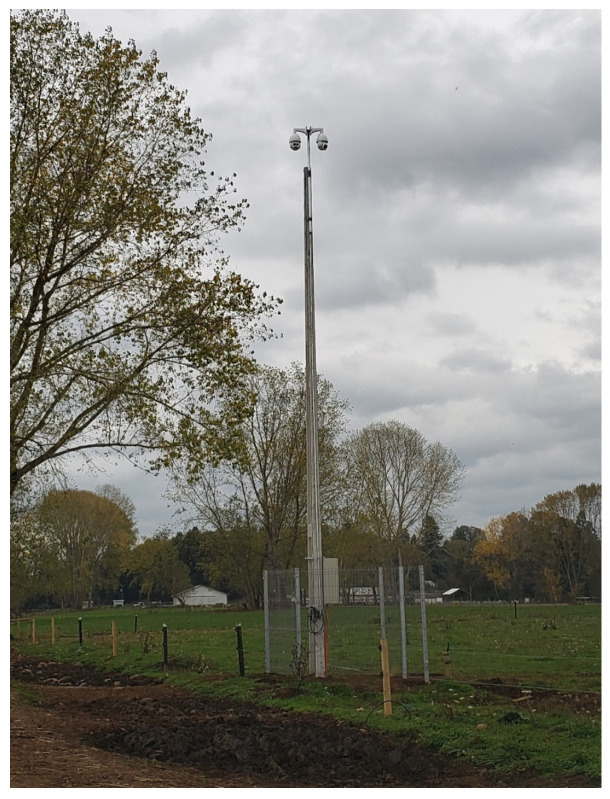
PTZ cameras located in the Maquehue Experimental Farm.

The cameras can be seen at the top of the pole in Figure 4.

### 2.2. Available Data

As part of the Fondecyt 1220178 project, data have been collected using IoT collars installed on the necks of cows at the Maquehue Experimental Farm of the Universidad de La Frontera since May 2022. These collars store information locally on microSD cards, and every two weeks, during the battery replacement cycle, the data are transferred to Google Drive and the project server.

Additionally, video recordings are stored on the project server. These consist of one-hour excerpts of the cattle filmed by the continuously operating camera system.

Since 12 November 2022, motion signals have been systematically stored, accumulating 176 *RUNs* compressed in ZIP format, with a total volume exceeding 40 GB. Each *RUN* represents the motion data recorded by an IoT collar on a specific cow during a campaign, from device installation to battery depletion.

Data collected between 21 February 2023 and 30 October 2024 are analyzed to construct the presented database. Within each *RUN*, the data are organized into folders based on the measurement days, containing CSV (Comma Separated Values) files that segment the information into fragments of 10,000 samples per file. With a sampling frequency of 10 Hz, each file represents approximately 16.67 min of data.

### 2.3. Database Generation

#### 2.3.1. Data Processing

The data corresponding to measurements taken by the BNO055 IMU (linear acceleration, gyroscope measurements, magnetometer measurements, and orientation in the form of quaternions) and the MPU9250 IMU (linear acceleration, gyroscope measurements, and magnetometer measurements) are processed. To convert the units to the corresponding physical units—linear acceleration (m/s^2^) and degrees per second (°/s)—a linear scaling based on the sensitivity and resolution of the sensors is applied. This process is described using the following expressions:

(1) $$a=\frac{a_{RAW}\cdot Range_{a}}{2^{b_{a}}}\cdot 9.807$$

(2) $$\omega =\frac{\omega_{RAW}\cdot Range_{\omega }}{2^{b_{\omega }}}$$

where

- $a_{RAW}$ and $\omega_{RAW}$ represent the raw measurements of acceleration and gyroscope measurement obtained from the sensors.
- $Range_{a}$ and $Range_{\omega }$ correspond to the maximum ranges configured for the accelerometer and the gyroscope, respectively.
- $b_{a}$ and $b_{\omega }$ are the resolution bit numbers of the accelerometer and gyroscope’s analog-to-digital converter (ADC), respectively.
- The factor 9.807 converts the measurements from g to m/s^2^.

For this project, the specific sensor configuration is as follows:

- Accelerometer (Bosch BNO055): sensitivity of ±8 g with a resolution of 14 bits.
- Gyroscope (Bosch BNO055): sensitivity of $\pm 2000^{\circ }$/s with a resolution of 16 bits.
- Accelerometer (InvenSense MPU9250): sensitivity of ±16 g with a resolution of 16 bits.
- Gyroscope (InvenSense MPU9250): sensitivity of $\pm 2000^{\circ }$/s with a resolution of 16 bits.

The acceleration measurements are also transformed to the WF. A rotation matrix derived from the quaternions provided by the BNO055 IMU is used to achieve this. This transformation ensures that the data are aligned with the terrestrial reference frame.

Transforming accelerations from the BF to the WF is described in [22].

#### 2.3.2. Movements of Interest

Most research on livestock activity classification develops behavioral models using machine learning techniques. A common approach is the “one vs. all” method, where each activity class is classified individually against all others, building a binary model for each activity. This method simplifies multivariate classification problems by breaking them down into multiple binary problems, making it easier to identify specific livestock behaviors, such as grazing, walking, and resting.

In this work, the “one vs. all” approach is applied. Two classes are separated to identify active mounts: one corresponding to active mounts and the other to movements that are not mounts. Therefore, it is necessary to label other types of events. The most typical ones observed in the video are selected.

The movements to be labeled and their descriptions are as follows:

- Active mounting: The cow mounts another cow, starting when the cow pushes itself up onto the other and ending when it dismounts from the other cow’s back.
- Walking: The cow moves from one location to another without remaining still, with regular and coordinated leg movements. It begins with the first step and ends with the last.
- Resting: The cow remains at rest, lying on the ground, without making significant movements.
- Grazing: The cow feeds on grass, starting when it lowers its head to consume it and ending when it raises its head after finishing.
- Head nodding: The cow performs repetitive head movements, either up and down or side to side, usually as part of social or exploratory interactions.

This allows for a classification between the mount and non-mount classes.

#### 2.3.3. Movement Labeling

Data labeling is a key process in constructing datasets in any field. High-quality, diverse, and well-labeled data are essential for training robust and generalizable machine learning models that can perform effectively in real-world scenarios [23].

A manual labeling method is carried out, which involves direct observation and manual annotation of the movements observed in each cow. Other authors have used this method to train livestock behavior classification models using classification algorithms, as in [13,24].

This database contains data from both WF and BF reference systems, allowing the study of the impact on the performance of automatic classification models when trained with data from different reference frames.

The labeling process proceeds by defining the movements to be labeled and the signals captured by the IMUs already processed. This process involves analyzing the video signals stored on the project server, visually identifying the movements of interest, and manually marking the start and end of each movement in the signals captured by the IoT collars.

Accurate synchronization between the video recordings and the signals measured by the IMUs is essential to ensure proper labeling. Due to technical issues in some collars, such as small restarts, a few seconds delay between both systems is common. This delay must be manually adjusted during the analysis, aligning specific events observed in the video with characteristic patterns in the signals captured by the collars, such as significant changes in acceleration.

The labeling process is entirely manual, making it a highly demanding task in terms of time and attention. Each movement must be identified and precisely delineated to ensure the generated database is accurate and reliable—variable, reflected in the labeled events having non-constant durations. Two research assistants participated in this project and performed the movement labeling. A veterinarian supported the labeling process by holding periodic meetings with the labeling team to validate each record in the database.

Active mounting is significantly more complex than movements labeled as walking or grazing, which are easier to observe in video. This is because it is a brief event performed by the cows and requires several conditions to be met: the cow performing the mount must wear a collar, and the camera must be correctly focused on that specific cow (which was a challenge in older videos where camera tracking was not perfected), among other factors. The cow’s mount must be observed in the video records, and the IMU signals must be noise-free and synchronized with the video record.

The labeling involved video analysis from approximately 200 h of footage recorded between 2022 and 2024, covering eight Holstein Friesian cows. The cameras used were fixed-position PTZ models (IP DAHUA SD6AL230F-HNI) with 500 m range and night vision, configured for automatic tracking using real-time GPS data from the IoT collars. This automated camera control system was developed and evaluated in previous stages of the project, as described by Muñoz et al. [21] and Pinilla and Muñoz [25]. The observers reviewed the footage manually and recorded each behavior event’s start and end times, the cow ID, and behavior type in structured Microsoft Excel spreadsheets. These annotations were then used to align each labeled segment with the corresponding sensor data to build the database.

The final result is a labeled signal in which each time segment has the corresponding class assigned: active mount, walking, resting, grazing, or head nodding. This labeling is essential for generating the database and subsequent feature extraction and supervised classification model training.

#### 2.3.4. Database Structure

**Figure 5:**
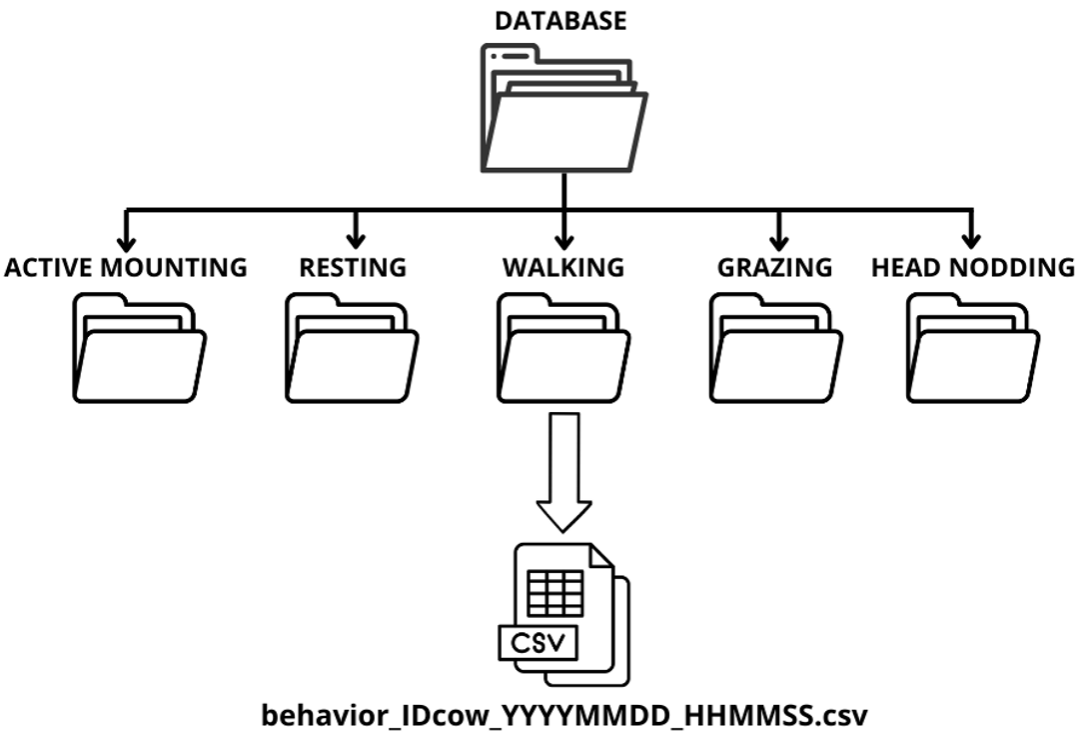
Database structure.

Figure 5 shows the database structure, where each type of movement has a specific folder. Within these folders are CSV files containing the signals corresponding to each movement. The duration of the signals ranges from 7 to 20 s for most movements. However, grazing signals can last up to 3 min, as cows eat continuously for several minutes. Since the sensors operate at a sampling frequency of 10 Hz, each file contains between 70 and 200 records for shorter movements or up to 1800 for grazing, depending on the signal’s duration. The name of each CSV file includes key information, such as the type of movement, the cow’s ID (a number assigned to each animal for identification in the field), and the date and time of the signal’s start.

The decision to organize the database in a structured collection of CSV files was made to prioritize simplicity, portability, and ease of use. This format allows researchers to directly access, inspect, and process the inertial signals without requiring a database management system or additional infrastructure. Furthermore, CSV-based organization provides flexibility for feature extraction and integration into machine learning pipelines using widely adopted tools such as Python (version 3.11.0). This design choice is aligned with practices adopted in other recent datasets on livestock behavior monitoring, such as the CowScreeningDB dataset, which focuses on lameness detection using accelerometer data stored in CSV files [4], and the dataset by Ito [26], published on Zenodo, which contains inertial sensor recordings from Japanese Black beef cows in grass fields and farm pens, also organized in CSV format.

Table 1 presents the columns of the CSV files, which correspond to the measurements obtained from the BNO055 and MPU9250 IMUs.

### 2.4. SVM Implementation to Evaluate the Database

#### 2.4.1. Proposed Features for Classification

The features used for model training, presented in Table 2, are based on those used in [13].

The features listed in Table 2 are extracted for each labeled event, specifically from the three axes of linear acceleration, the three axes of angular velocity measured by the gyroscope, and their corresponding magnitudes. That means a total of 112 features are extracted. Table 3 details the variables used for feature extraction.

The accelerations listed in Table 3 are extracted in two versions: one in the sensor’s BF and another in the WF. With this data, two independent classification models are trained: one based on accelerations in BF and another in WF. This allows for a comparison of the impact of the reference frame on the model’s performance, keeping all other conditions constant.

#### 2.4.2. Using the Database

The database is organized in folders, where each subfolder corresponds to a specific behavioral class (e.g., active mounting, walking, grazing). Each CSV file contains time-series data of inertial signals captured by the IoT collars, including three-axis acceleration and gyroscope measurements in the WF and BF (e.g., BNO055_ax_world, BNO055_ay_world, BNO055_az_world, GX, GY, GZ). To prepare the dataset for model training, a feature extraction routine is applied to each file. This involves reading the signals, optionally segmenting them using a fixed window (7 s), and computing time- and frequency-domain features from each signal axis and from the magnitude of acceleration and angular velocity. Each 7 s window is treated as a sample and labeled according to the associated behavior: class 1 for active mounting events and class 0 for all other behaviors such as walking, grazing, or resting. This setup defines a binary classification task. The feature extraction process converts each window into a numerical vector summarizing its main features. These vectors form the input matrix X, while their corresponding labels form the output vector y. The resulting dataset is then used to train and evaluate a binary classifier based on a Support Vector Machine (SVM), which is designed to distinguish between mounting and non-mounting behaviors. The following pseudocode (Algorithm 1) illustrates the whole process from reading CSV files to preparing the model input.
**Algorithm 1:** Processing inertial signals for binary classification of active mounting behavior.
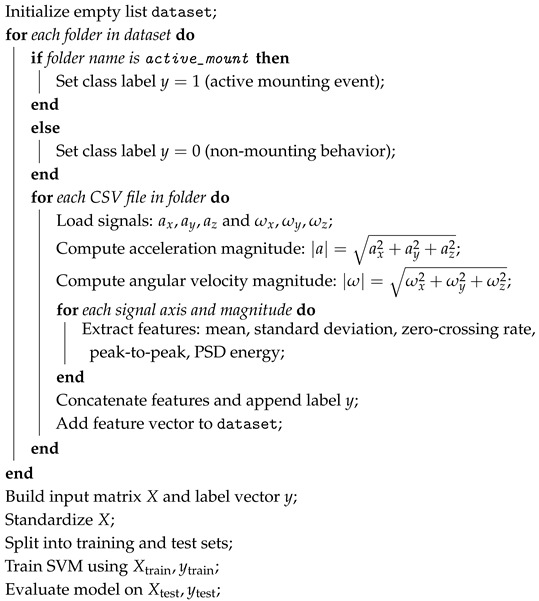


#### 2.4.3. Feature Selection

Feature selection is fundamental in improving a model’s performance and efficiency. Reducing dimensionality allows for the removal of redundant or irrelevant features, improving the model’s ability to generalize and preventing overfitting.

In Liang et al. [13], machine learning models for classifying bovine behaviors were trained using signals collected from IMUs. Two feature reduction techniques were used: permutation importance and Sequential Backward Selection (SBS). The authors reduced the feature space from 70 to 58, maintaining a high F1 Score, and the authors also show that reducing to 31 features was even possible, achieving a better balance between performance and efficiency. The best F1 Score achieved in this study was 0.87 using the 58 features, representing the maximum performance with the selected set. However, identifying a subset of 31 features maintained a remarkable F1 Score of 0.8506, demonstrating that high performance could be sustained despite eliminating many features.

Permutation importance in feature selection is crucial because it assesses the individual impact of each variable on the model’s performance, allowing for the identification of those that indeed contribute to improving classification ability. This method involves randomly altering the values of a feature while keeping the rest of the data intact, measuring the variation in the model’s F1 Score after the permutation. If altering a feature does not significantly affect this metric, it is considered dispensable, facilitating dimensionality reduction without compromising model effectiveness. Studies such as Altmann et al. (2010) [27] have shown that permutation is a robust strategy for estimating feature importance in machine learning models, providing interpretability and helping to prevent overfitting.

Sequential Backward Selection is an efficient technique for dimensionality reduction, as it progressively eliminates less relevant features until the optimal subset for the model is found. This method starts with all available features and, in each iteration, removes the one that least affects the model’s performance based on an evaluation metric, in this case, the F1 Score, which balances precision and sensitivity. By removing irrelevant or redundant variables, SBS helps improve the model’s ability to correctly detect the classes of interest, preventing the loss of relevant information for classification. Additionally, it reduces the risk of overfitting.

#### 2.4.4. Time Window

A fixed time window of 7 s is selected, representing the minimum duration observed in the labels. Although mounts have a variable duration, none are shorter than 7 s, ensuring that this window effectively captures the start of the activity without omitting relevant information.

#### 2.4.5. Classification Algorithm

The Support Vector Machines method is a supervised learning algorithm widely used for classification and regression. The main objective of SVM is to find the optimal hyperplane that separates the data classes in a high-dimensional feature space, maximizing the margin between the classes. This margin is the distance between the hyperplane and the closest points of each class, known as support vectors. SVM can handle nonlinear data using the kernel trick, which transforms the original data into a higher feature space, making linear separation possible. In its most basic formulation, SVM seeks to solve an optimization problem to maximize this margin and minimize the classification errors on the training points [28]. This algorithm assumes that the information is contained in a limited set of examples [29], making it suitable for situations with limited training samples. It has been implemented previously in the literature for this type of database; in [13], it was used for behavior pattern recognition in dairy cows.

This work uses the “one vs. rest” (OvR) classification technique. An SVM classifier is trained for each class independently, considering one class as positive against all others as negative. Specifically, active mount vs. other movements is classified.

Class imbalance is influential in building classification models, especially when the classes are imbalanced, such as active mounts vs. other movements. If one class is overrepresented, the model may develop a bias towards that class, reducing its ability to classify the minority class correctly. According to Chawla et al. (2002) [30], a common approach to address this issue is oversampling the minority class or undersampling the majority class. This work selects random samples from the “other movements” class to balance them with active mounts so that the model does not favor any classes and can better generalize to new data. This approach improves model performance.

The SVM classifier was implemented using the scikit-learn Python library (version 1.5.2) [31].

To train the classification model, the original dataset is split into 80% for training and 20% for validation. An SVM model with a linear kernel is used, and hyperparameter optimization is performed using the Grid Search method. Grid Search is an exhaustive search method that tests all combinations of predefined hyperparameter values to find the configuration that yields the best performance, based on a scoring metric. In this study, the regularization parameter C was optimized using the GridSearchCV module from the scikit-learn Python library, aiming to maximize the F1 Score through cross-validation.

Additionally, to assess the model’s performance and reduce the risk of overfitting, Repeated Stratified K-Fold cross-validation is applied. Specifically, a 5-fold cross-validation is repeated 20 times, resulting in 100 training iterations. The performance metrics are calculated for each 100 iterations and then averaged, ensuring that the final reported metrics reflect the overall performance across all training *RUNs* rather than a single evaluation. Repeated Stratified K-Fold cross-validation ensures that each fold contains a proportional representation of both classes, preventing the creation of folds with only one class. This stratified approach maintains the class distribution from the original dataset.

The performance of classification models should be evaluated using quantitative metrics that measure their effectiveness in predicting the correct classes. The most commonly used metrics are accuracy, precision, recall, and F1 Score. Accuracy measures the percentage of correct predictions over the samples evaluated. Precision represents the proportion of true positives to all instances classified as positive, that is, how reliable a positive prediction is. Recall measures the model’s ability to identify all positive instances within the dataset correctly. Finally, the F1 Score is the harmonic mean between precision and recall, balancing both metrics. The following equations formally define these metrics:

(3) $$\mathrm{Accuracy}=\frac{TP+TN}{TP+TN+FP+FN}$$

(4) $$\mathrm{Precision}=\frac{TP}{TP+FP}$$

(5) $$\mathrm{Recall}=\frac{TP}{TP+FN}$$

(6) $$F1=2\cdot \frac{\mathrm{Precision}\cdot \mathrm{Recall}}{\mathrm{Precision}+\mathrm{Recall}}$$

where TP represents the true positives, TN the true negatives, FP the false positives, and FN the false negatives. These metrics allow for evaluating the model’s performance in different scenarios and facilitate comparing different classification approaches.

#### 2.4.6. Statistical Analysis

With the 100 iterations, a statistical analysis is used to verify if there exists a significant difference between the F1 Score means of the SVM trained with the WF and BF. The Shapiro–Wilk and Levene tests validate the distributional assumptions of the F1 Score normality and variance homogeneity. Depending on the normality results, either the Student’s *t*-test is applied or the Mann–Whitney test is used without normality. The same analysis was repeated when the model was trained with SBS to reduce the feature space using the 100 iterations of the dataset. The JAMOVI (version 2.6) software is used for the analysis [32].

## 3. Results

### 3.1. Dataset

**Figure 6:**
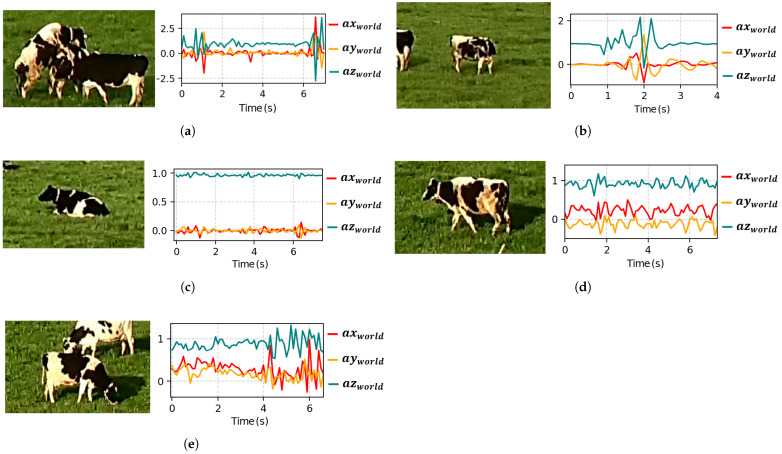
Screenshot from the video and acceleration record along the three axes (x, y, z) in the WF of the following: (**a**) cow mounting another; (**b**) cow head-nodding; (**c**) cow resting; (**d**) cow walking; (**e**) cow grazing.

Figure 6 present examples of labeled signals with the reference frame in the WF. On the left side of each figure, an image captured from the video corresponding to the event is shown, while on the right, the acceleration signals along the three axes (x, y, z) in WF are illustrated. The vertical axis in the figures represents acceleration measured in G units.

In Figure 6a, the onset of a mount can be observed, characterized by a first peak in the three acceleration axes. Subsequently, the central phase is distinguished, where the cow remains immobile over the cow accepting the mount, followed by a second peak associated with the cow falling to the ground floor.

Figure 6b displays a head-nodding event, highlighting the corresponding variations in the accelerations across the three axes. On the other hand, in Figure 6c, a cow resting on the ground is shown. In this case, the signals reflect minimal activity associated with small movements of the cow’s neck. Additionally, it is observed that the acceleration along the Z-axis is always close to 9.807 m/s^2^, as this axis is always aligned with the acceleration due to gravity when using the WF.

Figure 6d shows the signal of a walking cow, where activity is observed along the three axes, with greater intensity in the x and y axes. The signals display a continuous pattern consistent with the rhythmic nature of walking. Finally, Figure 6e presents a more erratic signal than walking, as grazing involves short but vigorous movements associated with chewing and grass consumption.

Table 4 summarizes the number of recorded CSV archives per behavior, along with their total and average durations (HH:MM:SS). Grazing had the longest total duration (01:37:46), while active mounting had the shortest (00:03:19 across 21 archives).

### 3.2. SVM Classification Model Considering All Proposed Features for Training

Table 5 presents the performance metrics of the model in classifying active mounts versus other movements. The model was trained using the 112 features proposed in Table 2, extracted from the variables defined in Table 3. This comparison analyzes the results obtained by employing accelerations in both the BF and WF. The best hyperparameter found using the Grid Search method with a linear kernel was C = 0.1.

An improvement in all metrics is observed in Table 5 when using accelerations in WF compared to BF.

Table 6 presents the descriptive statistics of the F1 Score obtained using the 112 proposed features in both the BF and WF. It can be observed that the model based on the WF achieves a mean F1 Score of 0.886 and a median of 0.889, both higher than those obtained in the BF (0.786 and 0.800, respectively). This suggests that transforming the signals to the terrestrial reference frame improves the classification model’s performance, particularly in this case, where the same 112 features are used for both reference frames.

On the other hand, the standard deviation remains similar in both cases (0.148 in Body and 0.132 in World), indicating that the choice of reference frame does not significantly affect the variability in the model’s performance.

**Figure 7:**
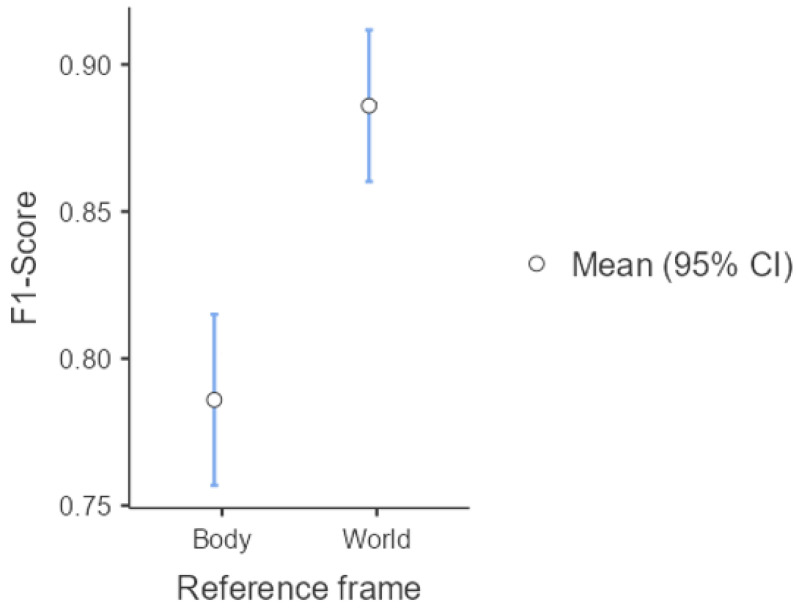
F1 Score comparison between BF and WF reference systems considering the 112 proposed features. Circles represent the mean values with 95% confidence intervals.

Figure 7 complements these findings by providing a visual representation of the 95% confidence intervals, along with the mean and median values. Notably, the median is closer to the mean in the WF, suggesting lower asymmetry in the F1 Score distribution.

Levene’s variance homogeneity test yielded a *p*-value of 0.428, exceeding 0.05. This indicates insufficient evidence to reject the null hypothesis, meaning the group variances do not significantly differ. Therefore, the assumption of homogeneity of variances holds in this analysis.

The results of the normality tests using the Shapiro–Wilk criteria applied to the indices indicate that F1 Score validation index does not follow a normal distribution (*p* < 0.001), justifying the use of non-parametric statistical methods for further analysis.

The Mann–Whitney U test (Table 7) indicates a significant difference between the two compared groups (BF vs. WF) with a *p*-value less than 0.001, suggesting that the distributions of the F1 Scores are not equal. Additionally, the effect size, measured using the rank biserial correlation, was 0.416, indicating a moderate relationship between the differences in F1 Scores between the two groups. These results show that the distributions of the F1 Scores are significantly different, with a moderate effect size reflecting a notable, though not exceptionally large, difference.

### 3.3. SVM Classification Model Using SBS-Selected Features

The features selected using SBS for each reference frame are shown in Table 8, selecting 13 features for WF and BF. After applying feature reduction, the results shown in Table 9 were obtained.

Feature reduction using SBS allowed for a more dimensionally compact feature set representation without compromising model performance. It increased the F1 Score by 12.9% in the BF and 1.1% in the WF. Thirteen features were selected for each reference frame.

Mounting and head-nodding behaviors display similar short-duration vertical motion patterns in their inertial signals, which may lead to occasional classification errors between these two classes. When we validated the classification errors using our trained model—specifically the one trained with World Frame (WF) data and feature selection via Sequential Backward Selection (SBS)—focusing solely on mounting and head-nodding data, we found a 39.5% misclassification rate. This misclassification rate is notably higher than that observed for other behaviors (see Table 10), and can largely be attributed to the vertical motion characteristics shared by both mounting and head-nodding events.

Table 11 presents the descriptive statistics of the F1 Score obtained after applying feature selection using SBS in both the BF and WF. The results indicate that the model trained in the BF achieves a higher mean F1 Score (0.915) than the WF (0.891). Additionally, the median in the BF reaches its maximum possible value (1.00), while in the WF, it remains at 0.889. These results suggest that, after feature reduction, the classification performance is slightly better when using the BF.

Regarding variability, the standard deviation is lower in the WF (0.094) compared to the BF (0.107), indicating more consistent model performance across different data samples in the WF.

**Figure 8:**
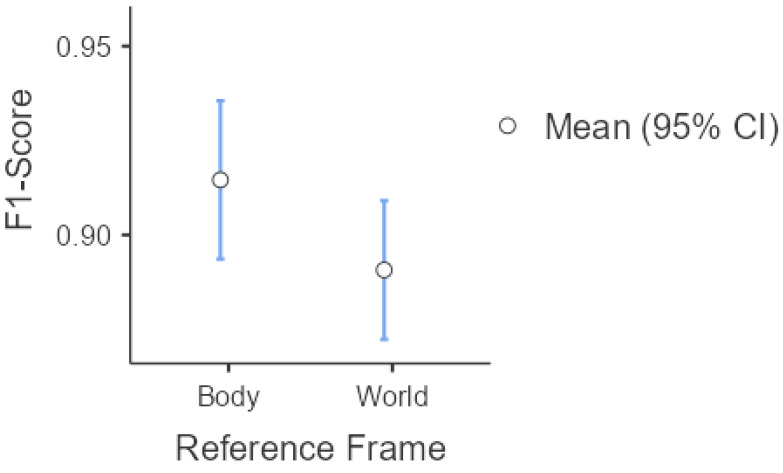
F1 Score comparison between BF and WF reference systems using the features selected by the Sequential Backward Selection (SBS) method. Circles represent the mean values with 95% confidence intervals.

Figure 8 visually represents the 95% confidence intervals, along with the mean and median values. Notably, in the BF, the median is considerably higher than the mean, suggesting a positively skewed distribution of the F1 Score. In contrast, the WF exhibits a median closer to the mean, indicating a more symmetrical distribution.

The F1 Score distribution does not fulfill the normality assumptions (*p*-value < 0.001) and the homogeneity assumptions (*p*-value = 0.002), so the nonparametric analysis is performed. Table 12 presents the descriptive statistics of the Mann–Whitney U test with their 95% confidence intervals when comparing means of BF and WF (case SBS). It is observed a statistically significant difference (*p*-value = 0.04) between the mean values of F1 Score for both groups (BF and WF), but with a small size of the effect (rank biserial correlation = 0.161). These results show that the distributions of the F1 Scores for both groups are slightly different when using SBS to select the features for the SVM.

## 4. Discussion and Conclusions

This study presents the development of a public database for detecting active mounts in cattle using IoT collars with inertial sensors and a reference frame in WF and BF. The generation of this database involved the collection, processing, and manual labeling of inertial signals obtained in the Maquehue Experimental Farm. Additionally, a classification model based on SVM was implemented to assess the effectiveness of the data in detecting active mounts.

The data captured in this project comes from free-grazing cattle, unlike related works [12,13,14,17], which have been conducted in confined environments. This represents a different environment where the cows live, transitioning from a more controlled and smaller space to larger, free-grazing systems. These conditions are less controlled and more representative of real-world dairy farms in countries like Chile. Additionally, signal capture is less invasive since the cameras are mounted on poles and do not directly interfere with the animals’ behavior, allowing them to act naturally. The database developed comprised 415 labeled behaviors (in five categories), with a total duration of 3:00:03, including 21 active mountings.

Previous studies, such as the one by Ismail et al. [4], have focused on detecting lameness in dairy cows using CowScreeningDB. This public database includes data from cows in confined environments. While this dataset includes information from multiple cows and various behaviors, it does not specifically address active mounts. Furthermore, this study expands upon previous databases from this project, such as [18], by incorporating acceleration data in both the WF and BF, enhancing the analysis and classification of bovine movements.

Similar studies by Liang et al. [13] and Peng et al. [12] implemented SVM and other classifiers such as Random Forest and XGBoost, achieving F1 Scores between 0.85 and 0.94 for specific behaviors like walking, resting, and feeding. In this work, active mounts are analyzed, being a more complex behavior. The mean F1 Score obtained in this study with an SVM is comparable, achieving 0.92 for active mounts when applying SBS reduction in features obtained on the BF.

The results from training the model with all the proposed features show that using the WF improves performance compared to the BF, achieving a mean F1 Score value of 10% higher, with a statistically significant difference.

This may be because measurements in the WF are more consistent and less susceptible to variations caused by sensor orientation. In BF, accelerations directly depend on the attitude estimation of the IoT collar’s IMU, which can introduce variability in the signal and affect the model’s accuracy. In contrast, accelerations in WF are referenced to a fixed frame of reference, allowing for a more stable representation of movements and, consequently, better discrimination between active mounts and other types of movement.

After applying the SBS technique to reduce the features for training the SVM, the difference between the means of the F1 Score obtained with the training group in the WF and the BF vanished. This is suitable because the IMU’s magnetometer needs to be recalibrated to obtain a good attitude estimation. This enables the coordinate rotation to translate the IMU’s accelerations from the BF to the WF.

However, it is noteworthy that the amount of active mount data in the training set is relatively low. This could introduce some bias into the model, making the obtained results not entirely representative. Although the observed improvement suggests that WF provides more helpful information for classification, the model’s performance may still be limited by the availability of data, which could affect its generalization to new scenarios.

External validation remains a critical challenge for sensor-based livestock technologies, as highlighted by Gómez et al. [33]. If the proposed system is deployed in other farms, it is relevant to compare the precision of automatic mount detection in free-grazing cattle, concerning different environmental conditions or cattle breeds.

Despite the progress in developing the database, some limitations must be considered. Firstly, the number of labeled active mount events is relatively low compared to the other classes. Specifically, 21 labeled active mount events out of 415 labels make up a small portion of the dataset. Therefore, future research should focus on increasing the number of labeled events and the number of cows under study to improve the model’s generalization.

In this study, hundreds of hours of video recordings had to be reviewed to manually identify and label active mounting events, primarily because we aimed to analyze the cow’s behavior in a natural environment. This means that the cows were video recorded on 80 hectares when free grazing, and in a significant portion of the footage, the camera did not adequately focus on the cattle at the precise moment of the event. This limitation posed a considerable challenge in the construction of the dataset. However, recent advancements in automated cattle tracking techniques have been implemented [25], which are streamlining the manual labeling process and boosting the database’s expansion. With these improvements, the number of labels associated with active mounts and other specific movements keeps increasing daily, enhancing the dataset’s representativeness and overall quality for future research.

Future database versions will increase the amount of registers, and could also incorporate other cow-related behaviors in natural environments, such as increased activity, grazing, passive mounting, and others, to create a more comprehensive tool for detecting cattle behavior. Moreover, exploring additional classification techniques could further enhance the detection accuracy and diminish the processing power requirements.

Although a dedicated time-series database or relational database management system was not required for the current data scale and processing needs, we consider including such systems in future iterations of this project, especially as the database grows or a real-time feed becomes necessary.

Currently, the data acquisition process is partially automated. The IoT collars collect IMU signals and store them locally, while GPS and battery status are transmitted via LoRaWAN to a Grafana dashboard. The PTZ cameras are also automatically controlled using GPS data from the collars as a reference. However, some processes remain manual, including data transfer, video-labeling synchronization, and feature extraction. We plan to develop a data pipeline to further automate these tasks using Python scripts. This will significantly reduce processing time and improve the efficiency of incorporating new data into the repository. Planned improvements include the development of a user-friendly interface for the labeling team, automated synchronization between IMU signals and video events, and semi-automated feature extraction routines.

## 5. Patents

The database was submitted for copyright claiming to the Intellectual Rights Department of Chile, the office in charge of copyright and other related rights over the Chilean country’s literary, artistic, and scientific works.

The database obtained from this study is all rights reserved (Submission Registration Code: 7ks1y1 to the “Departamento de Derechos Intelectuales” (DDI), Chile). Holder: Universidad de La Frontera.

## Figures and Tables

**Table 1:** Structure of processed data CSV files.

Sensor	Variables	Unit of Measurement	Column No.
BNO055	Time (date and hour)	YYYY-MM-DD hh:mm:ss	1
	Linear acceleration [WF] (x,y,z)	m/s^2^	2:4
	Linear acceleration [BF] (x,y,z)	m/s^2^	5:7
	Gyroscope measurement (x,y,z)	°/s	8:10
	Magnetometer measurement (x,y,z)	−	11:13
	Quaternions (Q1,Q2,Q3,Q4)	-	14:17
MPU9250	Linear acceleration [BF] (x,y,z)	m/s^2^	2:4
	Gyroscope measurement (x,y,z)	°/s	21:23
	Magnetometer measurement (x,y,z)	−	24:26

**Table 2:** Proposed features for classification.

Proposed Feature	Domain
Mean	Time
Median	Time
Standard deviation	Time
Zero-crossing rate	Time
Peak to peak	Time
Sum	Time
Sum of absolute values	Time
Root mean square	Time
Average acceleration variation	Time
Skewness	Time
Kurtosis	Time
Time between maximum peaks	Time
Dominant frequency	Frequency
Dominant spectral density	Frequency

**Table 3:** Data used for feature extraction and its description.

Data	Description
ax	Acceleration component in the X axis.
ay	Acceleration component in the Y axis.
az	Acceleration component in the Z axis.
|a|	Magnitude of acceleration $\left(\sqrt{ax^{2}+ay^{2}+az^{2}}\right)$.
ωx	Angular velocity component in the X axis.
ωy	Angular velocity component in the Y axis.
ωz	Angular velocity component in the Z axis.
|ω|	Magnitude of angular velocity $\left(\sqrt{\omega x^{2}+\omega y^{2}+\omega z^{2}}\right)$.

**Table 4:** Number of archives for each behavior.

Behavior	No. of Archives	Total Duration	Average Duration
Active mounting	21	00:03:19	00:00:09
Walking	186	00:45:41	00:00:14
Resting	83	00:27:40	00:00:20
Head nodding	43	00:05:37	00:00:08
Grazing	82	01:37:46	00:01:11
Total	415	03:00:03	-

**Table 5:** Average performance metrics comparison between WF and BF using all the proposed features.

Metric	Percentage (WF)	Percentage (BF)
Accuracy	89.5%	79.4%
Precision	92.2%	81.3%
Recall	87.4%	79.9%
F1 Score	88.6%	78.6%

**Table 6:** F1 Score statistics in BF and WF using the 112 proposed features.

Reference Frame	Mean	Median	Standar Deviation
Body	0.786	0.800	0.148
World	0.886	0.889	0.132

**Table 7:** Descriptive statistics of the Mann–Whitney U test with their 95% confidence intervals when comparing F1 Score means of Body Frame and World Frame (case with the 112 proposed features selected).

				95% Confidence Interval	
	Statistic	*p*	Mean Diff.	Lower	Upper
Mann–Whitney U	2920	<0.001	0.111	0.139	0.0880

**Table 8:** Selected features in BF and WF after feature reduction.

Feature	BF Data	WF Data
Median	az, ax	-
Zero Crossing Rate	az, ax, ay	az, ax, ωx
Peak to Peak	az	ωy
RMS	az	|ω|
Skewness	az	-
Dominant Frequency	az	az
Average Time Between Peaks	az, ax	az, ax, ay, ωx
Standard Deviation	ax, ay	az
Mean	-	|ω|
Kurtosis	-	az

**Table 9:** Average performance metrics comparison between WF and BF using SBS.

Metric	Percentage (WF)	Percentage (BF)
Accuracy	89.1%	90.3%
Precision	85%	88%
Recall	95.8%	96.7%
F1 Score	89.7%	91.5%

**Table 10:** False positive rate of each non-mount behavior classified as *active mount*.

Behavior	Total Samples (n)	False Positives (n)	Confusion Rate (%)
Grazing	82	13	15.85%
Head Nodding	43	17	39.53%
Resting	83	9	10.84%
Walking	186	30	16.13%

**Table 11:** F1 Score statistics in BF and WF using the features selected by SBS.

Reference Frame	Mean	Median	Standard Deviation
Body	0.915	1.00	0.107
World	0.891	0.889	0.094

**Table 12:** Descriptive statistics of the Mann–Whitney U test with their 95% confidence intervals when comparing F1 Score means of BF and WF (case SBS).

				95% Confidence Interval	
	Statistic	*p*	Mean Diff.	Lower	Upper
Mann–Whitney U	4196	0.040	2.41 × 10^−5^	2.38 × 10^−6^	0.0890

## Data Availability

The original data presented in the study are openly available in Github at https://github.com/WASP-lab/db-cow-mounts (accessed on 20 March 2025).

## Abbreviations

The following abbreviations are used in this manuscript:

PLF	Precision Livestock Farming
IMU	Inertial Measurement Unit
SVM	Support Vector Machine
SBS	Sequential Backward Selection
PTZ	Pan-Tilt-Zoom
CSV	Comma Separated Values
BF	Body Reference Frame
WF	World Reference Frame
OvR	One vs. Rest
